# Novel Ionic Liquid with Both Lewis and Brønsted Acid Sites for Michael Addition

**DOI:** 10.3390/ijms12117438

**Published:** 2011-10-28

**Authors:** Xiaoyue Jiang, Weidong Ye, Xiaohua Song, Wenxin Ma, Xuejun Lao, Runpu Shen

**Affiliations:** 1 Zhejiang Medicine Co. Ltd., Xinchang Pharmaceutical Factory, Zhejiang 312500, China; E-Mails: shiningchemical@126.com (X.J.); shiningchemistry@126.com (W.Y.); songxiaohua110@126.com (X.S.); shingchemical@126.com (W.M.); shingchemistry@126.com (X.L.); 2 College of Chemistry & Chemical Engineering, Shaoxing University, Shaoxing 312000, China

**Keywords:** novel ionic liquid, Michael addition, Lewis and Brønsted acid sites

## Abstract

Ionic liquid with both Lewis and Brønsted acid sites has been synthesized and its catalytic activities for Michael addition were carefully studied. The novel ionic liquid was stable to water and could be used in aqueous solution. The molar ratio of the Lewis and Brønsted acid sites could be adjusted to match different reactions. The results showed that the novel ionic liquid was very efficient for Michael addition with good to excellent yields within several min. Operational simplicity, high stability to water and air, small amount used, low cost of the catalyst used, high yields, chemoselectivity, applicability to large-scale reactions and reusability are the key features of this methodology, which indicated that this novel ionic liquid also holds great potential for environmentally friendly processes.

## 1. Introduction

**Scheme 1:**
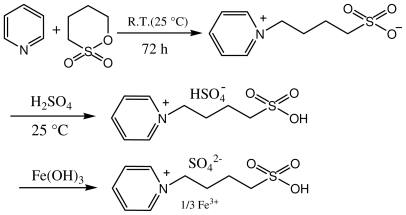
The synthetic route of the novel ionic liquid.

Acid catalysts are very important in chemical industries for the production of various chemicals [1]. Chloride and sulfonic alkyl groups functionalized ionic liquids were reported as environmentally friendly acidic catalysts due to the combination of the advantages of liquid and solid acids, e.g., uniform acid sites, easy separation and reusability [2]. Chloride ionic liquids were found to be very efficient for various reactions such as alkylation for the strong acidity, but these ionic liquids were very sensitive to water and could only be used in anhydrous conditions. Then Cole *et al*. [3] first reported on functional ionic liquid (FIL) with strong Brønsted acidity in 2002, which was stable to water. After that, the research and application of various –SO_3_H functionalized ionic liquids have received more and more attention [4,5]. The formation of carbon–nitrogen bonds by simple addition of amines to double bonds is the focus of increasing interest and widely used in organic synthesis owing to the importance of the resultant β-amino compounds [6]. These β-amino carbonyl compounds are versatile synthetic intermediates for the synthesis of a variety of biologically important natural products, antibiotics and are useful in fine chemicals and pharmaceuticals [7,8]. As we all know, both Lewis and Brønsted acid sites were very important for the acid-catalyzed reactions and the combination of Lewis and Brønsted acid sites in single ionic liquid would improve its catalytic activities. However, the water sensitivity of the Lewis acid sites would limit its application area in aqueous conditions. Here we present a novel ionic liquid with Lewis and Brønsted acid sites. In order to avoid the water sensitivity, which often occurred for the chloride ionic liquid, Fe^3+^ ion was introduced as the Lewis acid sites. The novel ionic liquid was synthesized through three steps. In the first step, zwitterion was obtained through the condensation of pyridine and 1,4-butane sulfonate. Then, equalmolar sulfuric acid and zwitterion were mixed together to form the homogeneous liquid phase. Some H^+^ ions were substituted by Fe^3+^ in the third step through the acid-base neotration reaction (Scheme 1). The catalytic activities of the novel ionic liquid were investigated through the Michael addition. The results showed that the ionic liquid was very efficient for the reactions with the average yields over 90% within several minutes.

## 2. Results and Discussion

### 2.1. Catalytic Procedure for the Conjugate Addition

The conjugate additions of various amines with alkenes under solvent-free conditions were investigated first (Table 1). The results showed that the reactions catalyzed by the novel ionic liquid went smoothly at room temperature within several min. The methylamine showed extremely high activity for all kinds of electron deficient alkenes with almost complete conversion within 5 min (entries 1, 5, 8). The novel ionic liquid with Fe^3+^ was stable to water and could be used in the methylamine aqueous solution. Also, the ionic liquid was not decomposed in the methylamine aqueous solution and could be reused without loss of activities (entries 1, 5, 8). The yields slightly dropped with the increasing carbon atomicity of the amines because of the steric hindrance (entries 1–4, 5–7, 8–10). The primary amines underwent the single substitution reaction under the reaction conditions. These results indicated the usefulness of the novel ionic liquid for the reactions and the reaction conditions are mild and not sufficient to cause double substitution reaction. The multi-substitution reactions could also be activated when more alkenes and high temperature were applied, then the double substituted products were obtained with high yields. As to the alkenes, the reactivity was affected by the electronic withdrawing groups (EWG) and the steric hindrance also had a certain effect on the reactions.

### 2.2. The Reuse of the Ionic Liquid

**Figure 1:**
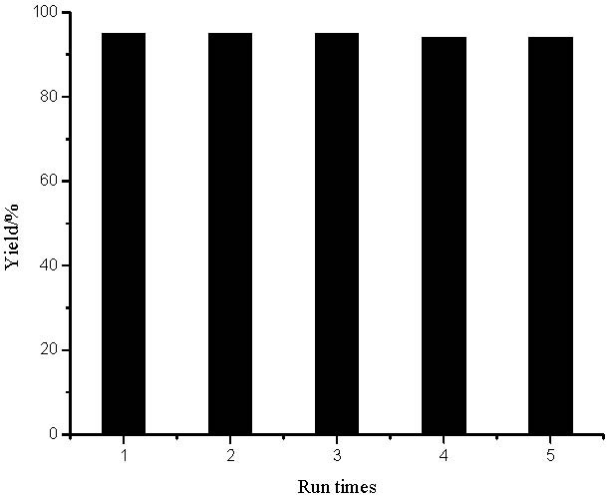
The reuse of the novel ionic liquid.

One property of the novel ionic liquid is the reusability. Thus, the recovery of the catalyst was very convenient. After reactions, the reaction mixture was extracted with ethyl acetate–ethyl ether = 1:1 and the lower phase, the ionic liquid, could be reused easily. The recovered activities were investigated through the reaction of cyclohexamine and methyl acrylate carefully (Figure 1). The yields remained unchanged even after the catalyst had been recycled five times.

### 2.3. The Chemoselectivity of the Novel Ionic Liquid

**Scheme 2:**
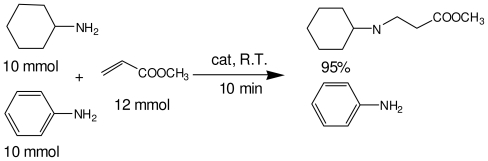
The chemoselectivity of the ionic liquid.

It is noteworthy that aromatic amines could not be transformed to the corresponding products under the same reaction conditions (Scheme 2). This result indicated that the present protocol could be applicable to the chemoselective addition of aliphatic amines in the presence of aromatic amines.

### 2.4. The Fe^3+^ Content on the Catalytic Activities of the Novel Ionic Liquid

**Scheme 3:**
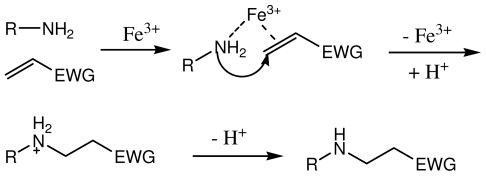
The probable catalytic procedure of the novel ionic liquid.

In order to investigate the effect of Fe^3+^ on the catalytic activities, ionic liquids with different Fe^3+^ content were utilized as the catalysts for the reaction of cyclohexamine and methyl acrylate (Table 2). It can be seen in Table 2 that both the Lewis acid site Fe^3+^ and the Brønsted acid site H^+^ were very important for the reaction. The catalytic activities were low with complete Lewis acid site Fe^3+^ or the Brønsted acid site H^+^ with long reaction time and low yield. The Fe^3+^ with high charges showed higher attraction for the reactants than the H^+^. The H^+^ attached to the double bond during the catalytic cycle, which was quite useful for the reaction. The catalytic activities reached the peak value when the Fe^3+^ content was 50% with the Lewis to Brønsted acid sites molar ratio of 1:1. Further increasing the Fe^3+^ content reduced the catalytic activities. These results indicated that Fe^3+^ and H^+^ ions cooperated with each other during the catalytic cycle and generated the higher activities than the single acid sites. Here, the probable catalytic procedure was demonstrated in Scheme 3. In the first step, Fe^3+^ cooperated with carbonyl group and diols, which made the hydroxyl groups attack the carbonyl groups more easily. Then the H^+^ attached to the double bond, which resulted in the release of the Fe^3+^. Both acid sites worked during the catalytic procedure, which made the novel ionic liquid more efficient for the reactions with less catalyst amount and higher yield.

### 2.5. The Comparative Study on the Catalytic Activities of the Different Catalysts

A comparative study on the catalytic activities of the novel ionic liquid with the traditional catalysts was carried out using the reaction of cyclohexamine and methyl acrylate (Table 3). From this study it can be concluded that the novel ionic liquid displayed even more activity than the traditional homogeneous catalysts; furthermore it adds the additional advantage of reusability. It clearly shows that this novel catalyst should be considered as one of the best choices for the economically convenient, user-friendly catalyst and for scaling up purposes.

## 3. Experimental Section

All organic reagents were commercial products with the highest purity available (>98%) and used for the reaction without further purification.

### 3.1. Synthesis of the Novel Ionic Liquid

The procedure for synthesizing the novel IL: pyridine (7.9 g, 0.1 mol) and 1,4-butane sulfonate (13.6 g, 0.1 mol) were mixed and stirred magnetically for 72 h at room temperature. Then, a white solid zwitterion was formed. The white solid zwitterion was filtrated and washed repeatedly with ether. After dried in vacuum (110 °C, 1.33 Pa), the white solid zwitterion was obtained in good yield (94%). A stoichiometric amount of sulfuric acid was added to the above obtained zwitterion and the mixture was stirred for 4 h at 60 °C to form the homogeneous liquid phase. Then, Fe(OH)_3_ (3.53 g, 0.033 mol) and 10 mL water were added to the liquid phase and the mixture was stirred until the solid was dissolve completely. Here the Fe^3+^ was introduced through the neotration reaction of Fe(OH)_3_ and HSO_4_^−^. Then, the water was removed by distillation and dried in vacuum (110 °C, 1.33 Pa). The product was formed quantitatively and in high purity with the forms of reddish-brown liquid (Melting point: 20–22 °C, viscosity: 82 × 10^−3^ Pa · s). The IL owned high thermal stability with the decomposition temperature above 200 °C. 1H NMR for the zwitterion (400 MHz, D_2_O, TMS): δ 1.74 (q, 2H), 2.11 (t, *J_H–H_* = 7.2 Hz, 2H), 2.90 (t, 2H), 4.60 (t, *J_H–H_* = 7.2 Hz, 2H), 8.01 (d, 2H), 8.49 (t, *J_H–H_* = 7.2 Hz, 2H), 8.80 (d, 2H). IR (KBr): 1037 cm^−1^ and 907 cm^−1^ (–SO_3_H), 1166 cm^−1^ (C–N), 3409 cm^−1^ (O–H). The element analysis: C: 32.6%; H: 4.2%; N: 4.2%; S: 19.4%. The results matched the structure in scheme 1 very well.

### 3.2. Conjugate Addition of Amines to Electron Deficient Alkenes

**Scheme 4:**
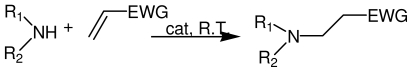
Conjugate addition of amines to electron deficient alkenes.

Typical procedure for the conjugate addition of amines (Scheme 4) was shown as follows: A mixture of amine (20 mmol), alkene (24 mmol) and the novel ionic liquid (0.04 g, 0.14 mmol) were stirred at room temperature for the certain time as shown in Table 1. The process of the reaction was monitored by GC analysis. The reaction mixture was extracted with ethyl acetate (2 × 20 mL) and the combined extract was dried over anhydrous Na_2_SO_4_ and evaporated to leave a crude product, which was separated by column chromatography using neutral alumina as stationary phase and petroleum ether/ethyl acetate mixture (95:5) as eluent to give the corresponding product.

## 4. Conclusions

The novel ionic liquid with both Lewis and Brønsted acid sites has been synthesized and its catalytic activities were carefully investigated through the Michael addition. The results showed that this novel ionic liquid was very efficient for traditional acid-catalyzed reactions and the Lewis and Brønsted acidic sites cooperated during the catalytic procedure. Operational simplicity, water-resistance, low cost of the catalyst used, high yields, applicability to large-scale reactions are the key features of this methodology. The combination of Lewis and Brønsted acid sites in single ionic liquid substantiates this catalyst holds great potential to replace tradition homogeneous acid catalysts in environmentally friendly processes.

## Figures and Tables

**Table 1:** The conjugate additions of various amines with alkenes.

Entry	Amines	Alkenes	Reaction Time/min	Yield, % a,b
1	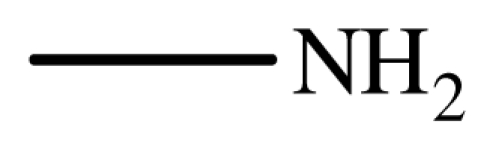	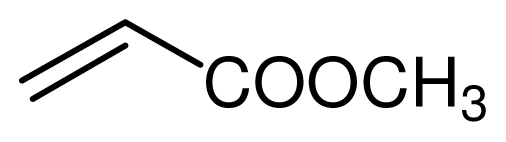	2	99
2	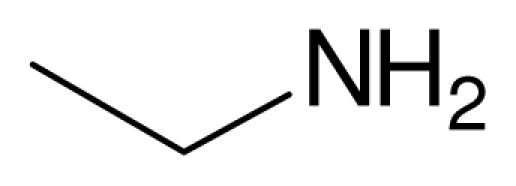	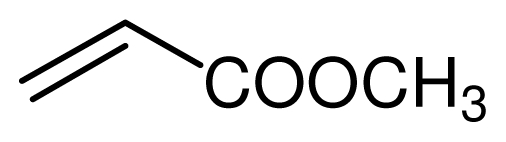	3	99
3	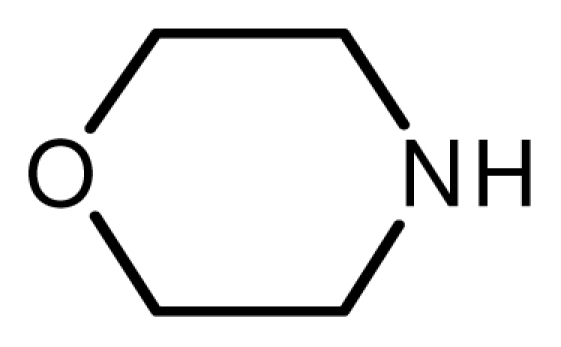	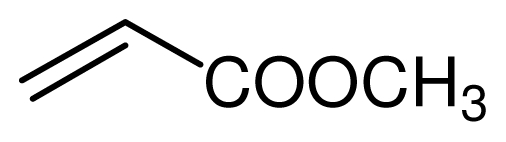	7	99
4	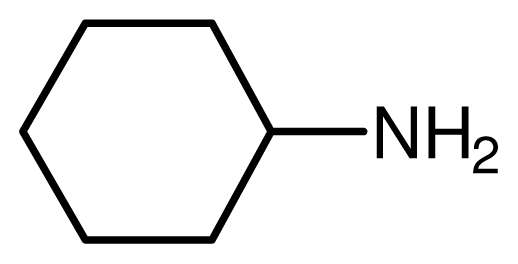	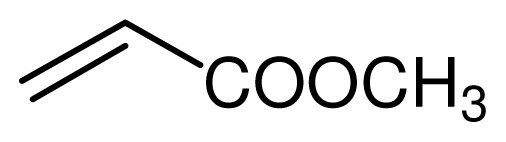	10	95
5	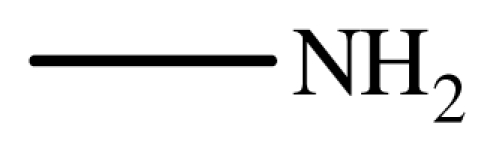	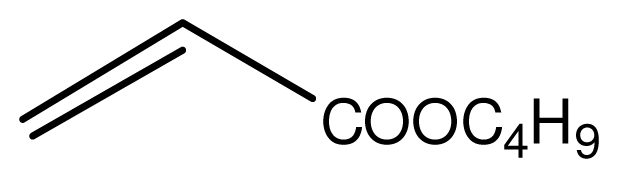	4	99
6	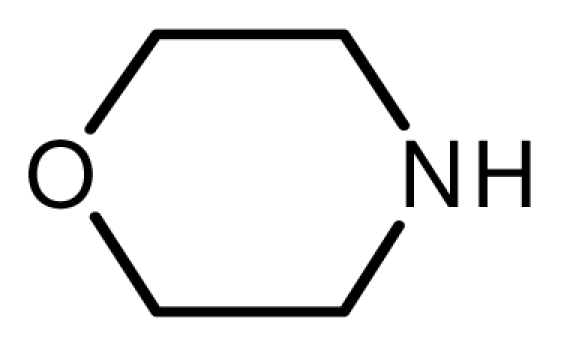	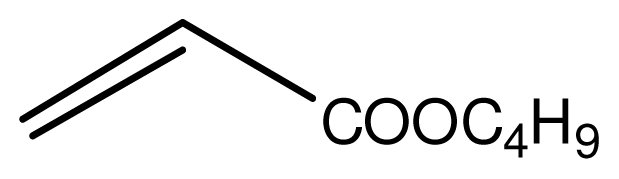	7	97
7	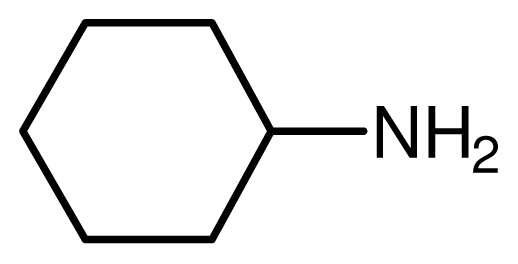	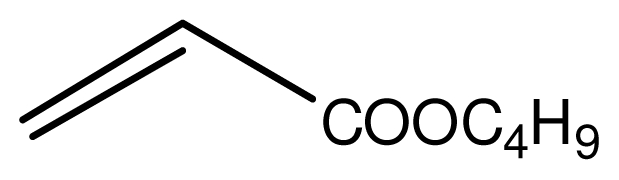	13	93
8	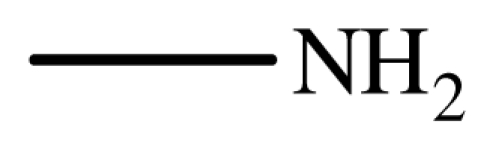	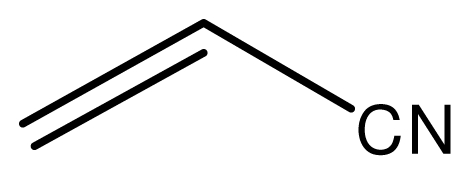	5	99
9	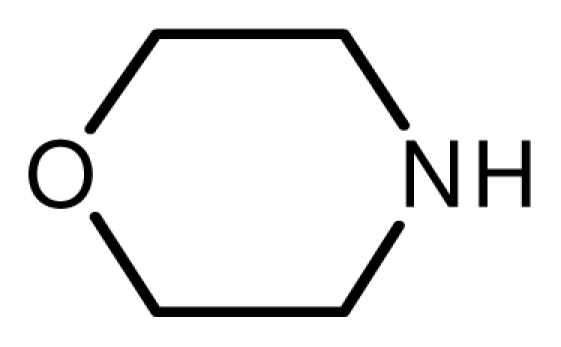	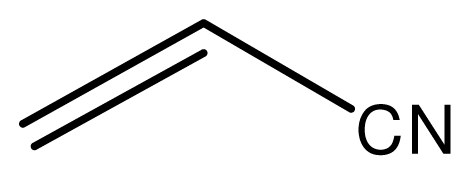	6	98
10	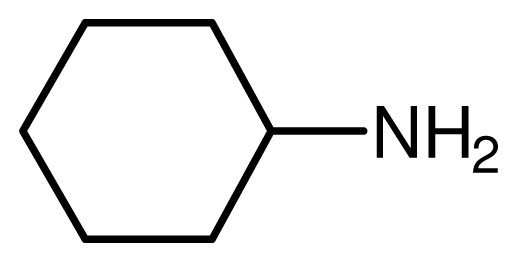	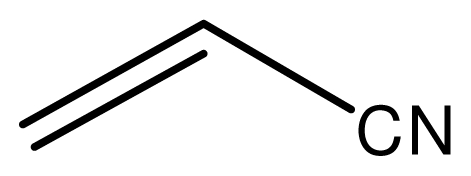	12	94

a The reaction conditions: amine 20 mmol, alkenes 24 mmol, ionic liquid (0.04 g, 0.14 mmol), R.T. (25 °C)

b The yield was estimated by GC analysis.

**Table 2:** The effect of the Fe^3+^ content on the catalytic activities.

Entry	Fe^3+^ content/% c	Catalyst amount/mmol	Reaction time/min	Yield/% a,b
1	0	1.00	25	90
2	25	0.50	15	92
3	50	0.14	10	95
4	75	0.75	20	93
5	100	1.20	30	90

a Reaction conditions: cyclohexamine 20 mmol, methyl acrylate 24 mmol, R.T. (25 °C);

b GC yields;

c Fe^3+^ content was measured as follows: n(Fe^3+^)/[n(Fe^3+^) + n(H^+^)] × 100%.

**Table 3:** The comparison of different catalysts.

Entry	Catalyst	Catalyst amount/mmol	Reaction time/min	Yield/% a,b
1	Novel ionic liquid	0.14	10	95
2	H_2_SO_4_	1.5	15	90
3	PTSA	2.0	12	92
4	BF_3_–OEt_2_	5.5	25	88
5	[mimbSO_3_H][HSO_4_]	1.5	15	91
6	ZnCl_2_	6.5	20	88

a Reaction conditions: cyclohexamine 20 mmol, methyl acrylate 24 mmol, R.T. (25 °C);

b GC yields.
